# Supplementary material for: A high-resolution mRNA expression time course of embryonic development in zebrafish
Source: eLife. 2017 Nov 16;6:e30860. doi: 10.7554/eLife.30860 (PMC5690287; doi:10.7554/eLife.30860)
Supplement: Supplementary file 6. [file elife-30860-supp6.zip › biolayout-clusters-files/Cluster132-genes.html]

Cluster132


# Cluster132: Genes

| Ensembl ID | Gene Name | Chr | Start | End | Biotype |
| --- | --- | --- | --- | --- | --- |
| ENSDARG00000092609 | aup1 | 14 | 5528300 | 5553350 | protein\_coding |
| ENSDARG00000076791 | cdk13 | 24 | 988778 | 1014423 | protein\_coding |
| ENSDARG00000040334 | mat2aa | 10 | 7850319 | 7862987 | protein\_coding |
| ENSDARG00000041072 | mier1a | 6 | 34842315 | 34855028 | protein\_coding |
| ENSDARG00000002949 | ppp6c | 5 | 31382945 | 31395248 | protein\_coding |
| ENSDARG00000007923 | ptpn1 | 8 | 28343617 | 28359157 | protein\_coding |
| ENSDARG00000073737 | suds3 | 5 | 14995134 | 15013561 | protein\_coding |
| ENSDARG00000056932 | tfip11 | 23 | 17922650 | 17939757 | protein\_coding |
| ENSDARG00000037966 | thoc6 | 3 | 32519983 | 32526722 | protein\_coding |
| ENSDARG00000026225 | whsc1 | 13 | 12861376 | 12898376 | protein\_coding |
